# Analyzing microglial phenotypes across neuropathologies: a practical guide

**DOI:** 10.1007/s00401-021-02370-8

**Published:** 2021-10-08

**Authors:** Marius Schwabenland, Wolfgang Brück, Josef Priller, Christine Stadelmann, Hans Lassmann, Marco Prinz

**Affiliations:** 1 Institute of Neuropathology, Medical Faculty, University of Freiburg, Freiburg, Germany; 2 Institute of Neuropathology, University Medical Center Goettingen, Goettingen, Germany; 3 School of Medicine, Technical University of Munich, Munich, Germany; 4 Department of Psychiatry and Psychotherapy, Klinikum Rechts Der Isar, Munich, Germany; 5 University of Edinburgh and UK DRI, Edinburgh, UK; 6 Neuropsychiatry and Laboratory of Molecular Psychiatry, Charité-Universitätsmedizin Berlin, Berlin, Germany; 7 DZNE, Berlin, Germany; 8 Center for Brain Research, Medical University of Vienna, Vienna, Austria; 9 Signalling Research Centres BIOSS and CIBSS, University of Freiburg, 79106 Freiburg, Germany; 10 Center for Basics in NeuroModulation (NeuroModulBasics), Faculty of Medicine, University of Freiburg, 79106 Freiburg, Germany

**Keywords:** Microglia, Microglia morphology, Microglia histology, Microglia immunohistochemistry, Microglia markers, Iba1, Human microglia, Microglia histopathology, Microglia phenotype

## Abstract

As extremely sensitive immune cells, microglia act as versatile watchdogs of the central nervous system (CNS) that tightly control tissue homeostasis. Therefore, microglial activation is an early and easily detectable hallmark of virtually all neuropsychiatric, neuro-oncological, neurodevelopmental, neurodegenerative and neuroinflammatory diseases. The recent introduction of novel high-throughput technologies and several single-cell methodologies as well as advances in epigenetic analyses helped to identify new microglia expression profiles, enhancer-landscapes and local signaling cues that defined diverse previously unappreciated microglia states in the healthy and diseased CNS. Here, we give an overview on the recent developments in the field of microglia biology and provide a practical guide to analyze disease-associated microglia phenotypes in both the murine and human CNS, on several morphological and molecular levels. Finally, technical limitations, potential pitfalls and data misinterpretations are discussed as well.

**Supplementary Information:**

The online version contains supplementary material available at 10.1007/s00401-021-02370-8.

## Introduction into the origin and function of microglia

More than 100 years ago, a dispute arose among two famous Spanish neuroscientists. Santiago Ramón y Cajal had predicted a third element (respectively, cell type) besides neurons and astrocytes [64]. Over time, it became evident that the third element comprised microglia and oligodendroglia. Since his tutor’s hypothesis lacked further specifications concerning features, morphology and functions of the third element, it was Pío del Río Hortega who first characterized microglia [50]. The controversy about the origin of the newly discovered cell type could not be settled at that time. For many decades after the dispute, little attention was paid to microglia cells. It is therefore not surprising that it took almost a century to finally clarify the origin of microglia cells. During primitive hematopoiesis in mice, c-kit^+^ stem cells in the extraembryonic yolk sac mature into CD45^+^ c-kit^−^ Cx3cr1^+^ macrophages [28]. Those cells invade the developing brain via the primitive bloodstream and give rise to definitive microglia [55]. Similar to circulating myeloid cells and tissue macrophages in the periphery, microglia have dual functions during non-inflammatory and inflammatory conditions [10]. In a non-inflammatory, healthy central nervous system (CNS), microglia were formerly believed to be quiescent and resting. On the contrary, microglial have motile processes and protrusions. They are constantly moving and thereby actively surveying their environment. During postnatal development, microglia are involved in axonal guidance and synaptic pruning, the physiological process in which abundant synapses are eliminated [49]. Therefore, the neuronal circuit maturation heavily relies on microglial involvement. Another function of microglia is to phagocyte any apoptotic debris, reducing proinflammatory cytokine secretion and minimizing tissue injury [34]. As a result, their exaggerated activation may also cause damage to the CNS [52].

Given that microglia can be both beneficial and detrimental to brain homeostasis and brain health, it has been shown that microglia are involved in the pathogenesis of a variety of diseases such as multiple sclerosis (MS) [36], Alzheimer's disease [41, 75], Parkinson's disease [77], autism spectrum disorder (ASD) [78] and even COVID-19 encephalitis recently [53, 54, 62]. Therefore, studying the underlying mechanisms of microglial activity potentially helps to better understand many neurological and neuropsychiatric disorders and may provide novel therapeutic approaches.

In addition, studying the microglial phenotype can also be interesting for researchers outside of the microglial community. As guardians of the brain, the cells rapidly react to any changes in brain homeostasis. In the event of brain pathologies, the microglial phenotype is certainly altered. Therefore, analyzing microglia can be a sensitive tool to check for CNS involvement in any given patient specimen or mouse model.

Within the past years, the microglia field and its technical possibilities have been evolving enormously. In this overview, we will summarize these developments and provide an easy point-by-point guide for assessing different microglial phenotypes.

## Murine versus human microglial cells: similarities and dissimilarities

Microglia have previously been studied in mammals including humans and rodents, but also in amphibians, reptiles, birds and annelids [16]. In all animals, microglia present themselves as CNS-resident cells with a ramified shape. In some species, microglia have more protrusions or a higher volume. Moreover, differences in microglia density are observed. Further examination of the cell transcriptome revealed a common microglial core gene expression pattern that is conserved across evolution. This core signature includes genes involved in microglia development (e.g. *Csf1r, Spi1*, *Irf8*), lysosomal markers (e.g. *Ctsb*) or genes that have previously been identified as “microglia-specific” since they are barely expressed in other (infiltrating) immune cells (e.g. *Tmem119*, *CD81*, *Sall1*, *Hexb* and *P2ry12*). In addition, some genes such as *Msr1* are predominantly expressed in primates. In contrast, the expression in rodents including mice and rats is extremely low. *Msr1* encodes for macrophage scavenger receptor 1 that is involved in amyloid beta (Aβ) processing. The gene is thought to be a risk locus for Alzheimer’s disease, as shown in genome-wide association studies. Microglia in the brains of rodents which are housed under specific-pathogen-free laboratory conditions show a stable homeostatic phenotype. In contrast, some microglial activation markers may be observed in the normal white matter of patients, devoid of any known neurological disease [76]. As an example, the major molecules involved in oxygen radical production, such as those of the NOX2 complex are already expressed in the normal brain and highly up-regulated in inflammatory or neurodegenerative diseases [76, 79], while in rodents their expression is restricted to a very small subset of microglia even under inflammatory conditions [40]. Another major difference is the profound iron loading of microglia in the aging human brain [23], which is associated with microglia senescence [68] and is virtually absent in the rodent brain. These examples highlight the limitations of animal models when examining microglia in the context of human diseases because they may not completely reflect the human disease pathogenesis.

## Non-invasive imaging techniques for microglia analyses

Physicians have a great interest in visualizing the activation states of microglia non-invasively in the human brain. In particular, positron emission tomography using microglia-specific radiotracers could help to clarify uncertain diagnoses or monitor the course of a disease such as multiple sclerosis.

Many chemical compounds targeting e.g. TSPO [74], P2X7 [30], CB2 receptor [44], COX-2 or CSF1R [24] have been developed in the past decades. The radiotracers all face similar problems. One major issue is the binding specificity of the compounds in vivo. On the one hand, polymorphisms in the target gene may determine the binding affinity [74]. On the other hand, unspecific binding potentially reduces the diagnostic power of the analysis. Moreover, the radiotracers’ targets are not necessarily specific for microglia cells. For instance, it has been shown that TSPO is also expressed by other cell types such as astrocytes or glial progenitor cells and thus cannot be seen as a microglia-specific radiotracer target. Moreover, a recent study could show that the findings from animal models do not always translate to the clinical context in humans [47]. Despite the fact that existing radiotracers need further improvement, the approach holds great promise for patients with (suspected) CNS pathologies. Even more, they allow to monitor disease activity longitudinally in chronic diseases, such as multiple sclerosis, and could thus be used as a paraclinical tool to monitor the effect of neuroprotective treatments [66, 69].

## Classical staining techniques for microglial cells

**Fig. 1:**
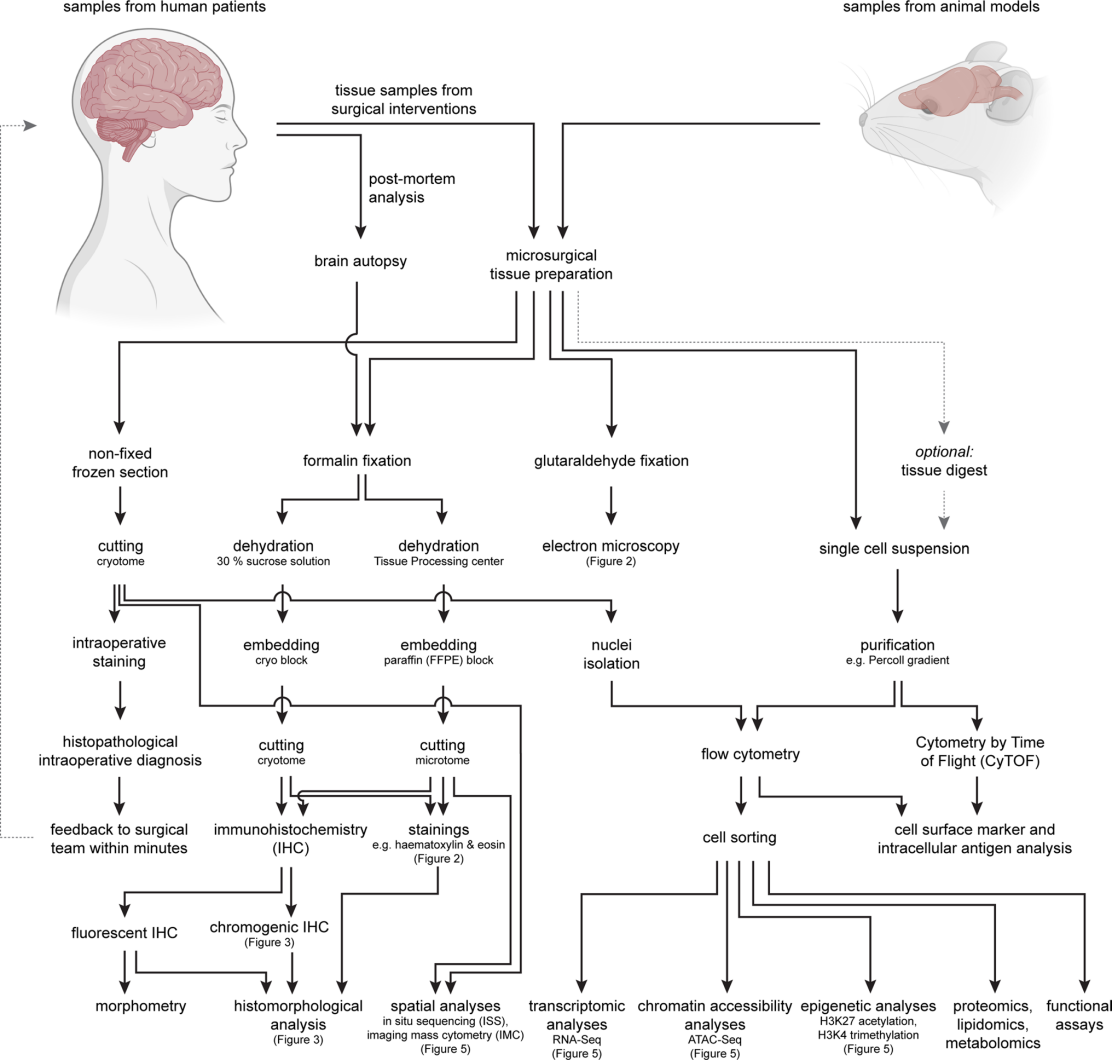
The workflow of tissue processing for the analysis of microglial cells using conventional and novel state-of-the-art technologies is depicted. The technical spectrum for the characterization of microglia cells and the necessary tissue pretreatment are shown

**Fig. 2:**
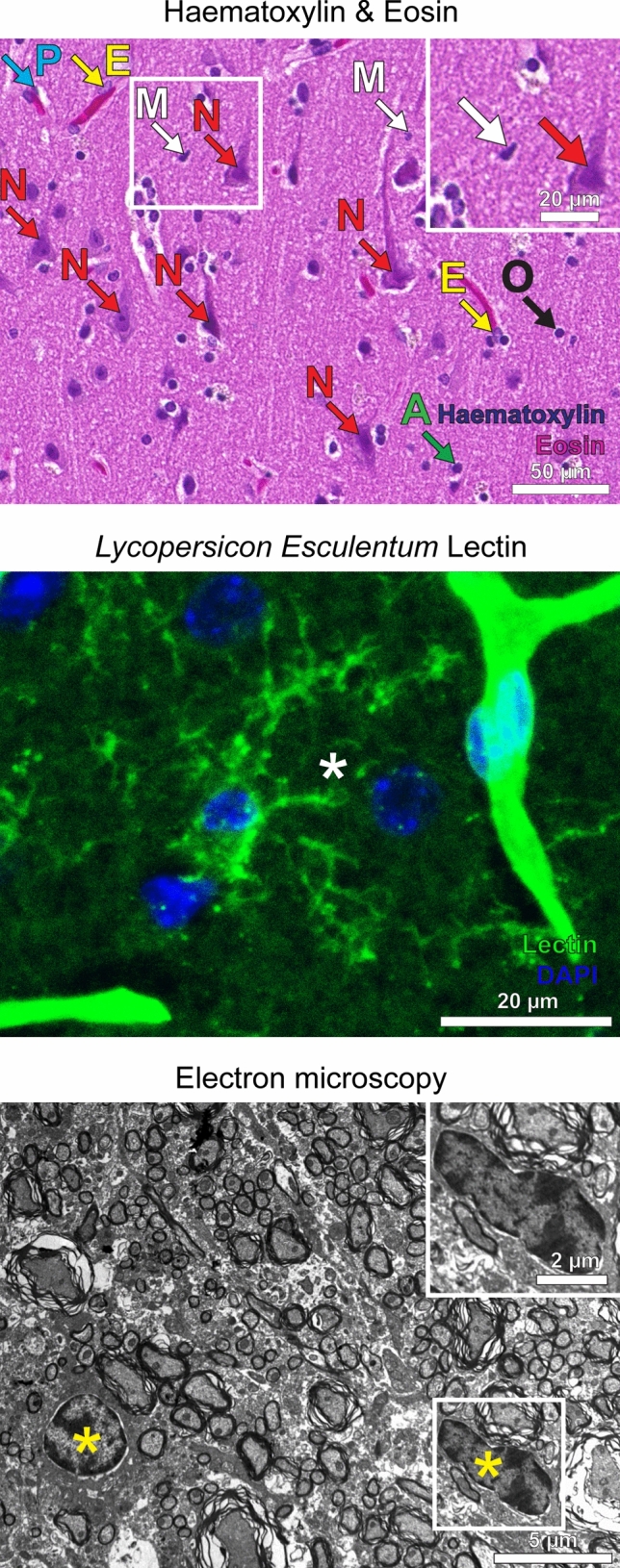
Visualization of microglia by using non-antibody-dependent staining techniques. **a** Different cell types including microglia can be identified in a Haematoxylin & Eosin section (H&E) of a healthy human cortex. Microglial nuclei (M) are small and dark, partially appear irregular and sometimes elongated with a “cigar-like” or “comma-like” shape. Perivascular macrophages (P) are located along blood vessels, in which endothelial cells (E) can be identified by their elongated nuclei. Arrows also point towards neurons (N), oligodendrocytes (O) and astrocytes (A). Scale bars: 50 µm and 20 µm (insert). **b** Lectins stain ramified microglia within the cortex of a mouse brain (asterisk). Cerebral vessels are stained as well (upper right). The staining with lectin (obtained from *Lycopersicon Esculentum*) is shown in green, DAPI in blue. Scale bar: 20 µm. **c** An electron microscopy image of a murine spinal cord is depicted. Microglia are typically small in size, partly have a “bean-shaped” nuclei (yellow asterisks) and only show little cytoplasm. Scale bars: 5 µm and 2 µm (insert)

The haematoxylin and eosin (H&E) stain is the most widely applied technique for routine histopathological examination of human tissue samples (Figs. 1, 2). Although the cytoplasm of microglia is hardly visible in H&E-stained specimens, microglial nuclei can be identified by their characteristic shape [15]. They are typically dark and relatively small in size. The partially occurring elongated nuclear shape had been termed “bean-shaped”, “cigar-shaped” or “comma-shaped” in the literature [14, 15]. Moreover, the contour of microglial nuclei partially appears irregular [15]. In the beginning of the twentieth century, different silver impregnation methods enabled Santiago Ramón y Cajal and his student Pío del Río Hortega to describe and characterize microglia.

Since the late 1950s, electron microscopy (EM) was used to determine the ultrastructure of microglia [42, 59, 60]. In EM, microglia appear rather small in size and may have a bent, “bean-shaped” nuclei [59]. The cytoplasm typically appears sparse and electron-dense [59] and frequently contains polymorphic electron-dense inclusions. Additional labelling, e.g. by gold-coupled antibodies, can help to identify the cells. Later, it has been shown that lectins, such as isolectin B4, that can be obtained from seeds of the African plant *Griffonia simplicifolia* or the tomato plant *Lycopersicon esculentum*, can be used for staining microglia cells [57, 67].

Nowadays, silver impregnations and lectins are hardly used anymore. They have almost completely been replaced by a more specific method, namely immunohistochemistry (Supplementary Fig. 1). Immunohistochemical reactions against the ionized calcium-binding adapter molecule 1 (Iba1) are routinely used to get a first impression of the microglia in a tissue sample. The cytoplasmic immunoreactivity nicely visualizes the cell shape and all processes. As described below, the morphology of the cell can provide insights about the activation status. Immunohistochemical reactions against human leukocyte antigen DR (HLA-DR) are commonly used in addition. Immunohistochemistry against macrosialin (CD68) reveals the degree of lysosomal activity. Since virtually all CNS pathologies involve microglia activation, the microglial phenotype alone can reveal whether a tissue sample must be considered pathological or not. Specifically, a tissue specimen with an entirely normal, homeostatic microglial phenotype excludes pathological CNS processes with almost complete certainty. Conversely, microglial alterations indicate CNS pathologies within the examined sample or even in the in situ CNS neighborhood. While this is obvious for young rodents housed under specific-pathogen-free conditions, the interpretation of microglial activation in the human brain can be more challenging. Non-neurological comorbidities and ongoing systemic therapies need to be taken into consideration.

## Microglia phenotypes in health and disease

**Fig. 3:**
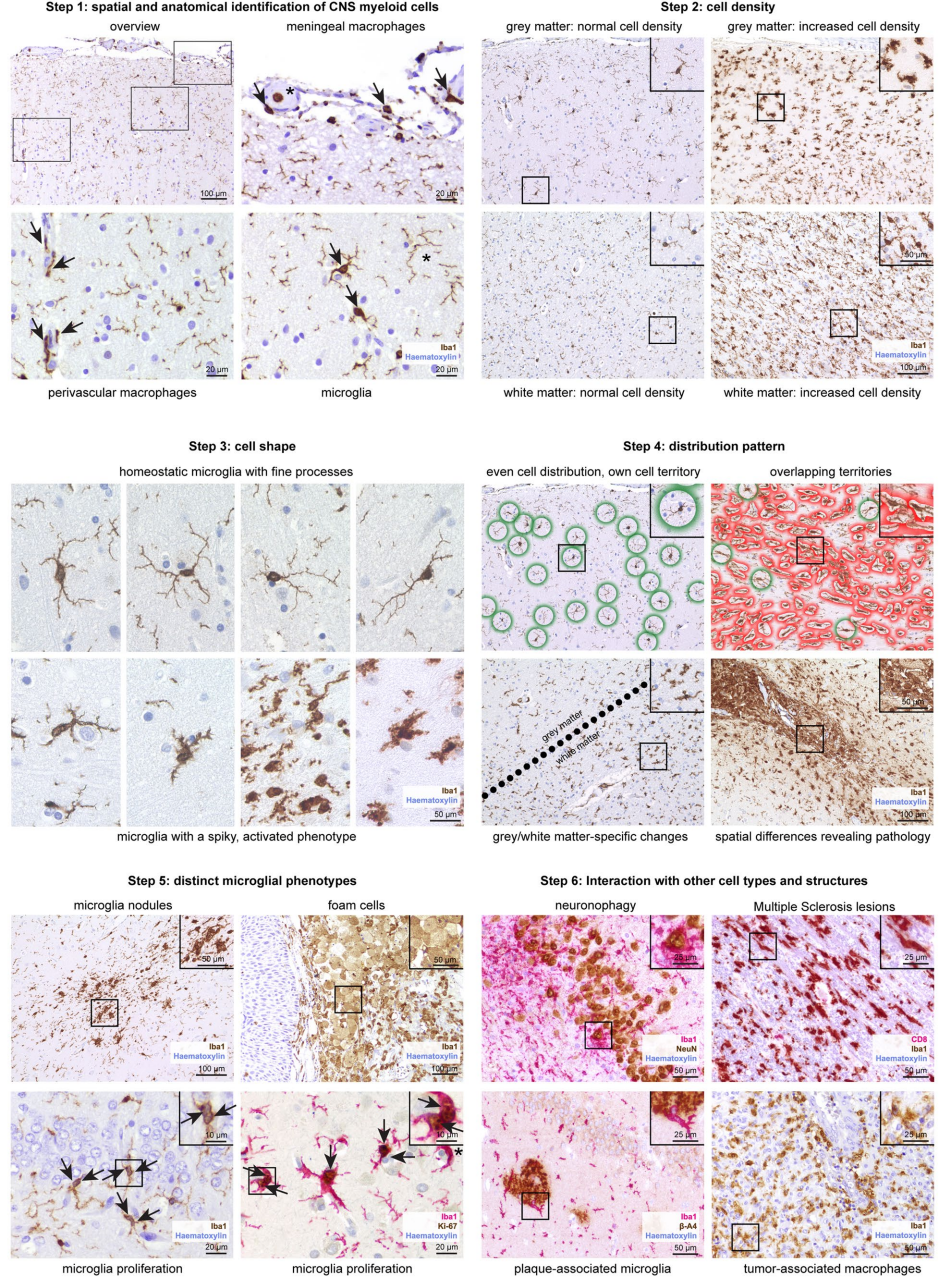
Immunohistochemistry-based assessment of microglia phenotypes across neuropathologies. Using Iba1 immunohistochemistry (brown), the following six-step standardized sequence is recommended for the analysis of microglia cells. In short, microglia cells and CNS-associated macrophages first need to be identified by their spatial distribution. Secondly, the cell density should be evaluated. The cell shape is of particular importance when analyzing the microglial phenotype. An uneven, irregular distribution pattern and microglia cell territories that are potentially overlapping might point towards local pathological processes within a tissue section. Next, the examiner should look for distinct microglia phenotypes, e.g. dividing cells or cells that underwent foam cell transformation. Lastly, a potential interaction with other cell types should be explored. Combined immunohistochemical reactions with two different markers are indicated. Counterstaining with haematoxylin (blue)

Using light microscopy, chromogenic DAB-based immunohistochemistry against Iba1 is a widely established method to study the morphological characteristics of microglial cells in both murine and human tissue specimens. We recommend the following standardized approach to assess microglial changes in CNS during homeostasis and perturbance (Fig. 3, Supplementary Fig. 2).

### Step 1: Identification of myeloid cells within the CNS

Iba1 is a reliable cytoplasmic microglial marker with a strong signal, labelling both cell bodies and processes (Fig. 3, Step 1, Supplementary Fig. 2a). Nevertheless, there is a major pitfall in using this marker, since Iba1 does not exclusively label microglia cells. It also marks other cell types such as perivascular or meningeal macrophages or even infiltrating myeloid cells such as monocytes. Due to their location within the meninges, meningeal macrophages can easily be identified. Distinguishing perivascular macrophages from microglia can be more challenging. Perivascular macrophages typically present with an elongated shape and unlike microglia without many processes. By nature, identifying vessels helps to find perivascular macrophages. With haematoxylin as counterstaining, vessels may show a lumen. Only if the vessel is cut transversally, a roundish shape might be visible. Longitudinally cut vessels show series of elongated nuclei and potentially an elongated lumen. In cortical biopsies, the course of the vessels is typically perpendicular to the cortical tissue surface, which can help to identify vessels and subsequently perivascular macrophages. After excluding meningeal and perivascular macrophages, the remaining parenchymal Iba1-labelled cells with processes are microglia. Notably, infiltrating monocytes would also be Iba1-positive. Although the cell shape may help to distinguish them from microglia to some extent, infiltrated monocytes may become ramified in the brain extracellular space [31].

### Step 2: Cell density

Microgliosis is defined as an elevated number of microglia cells (Fig. 3, Step 2). For better comparison, only cells with a visible soma/nucleus should be taken into account. Fine processes of microglial cells whose somata are not visible and most likely located in the consecutive tissue section should not be counted. A microgliosis is always a sign of ongoing pathology that is caused by microglial cell proliferation and/or myeloid cell infiltration. Unless a developing brain with a physiological microgliosis is examined, the observation of higher numbers of microglia cells alone proves that the tissue is not completely homeostatic or healthy. The microgliosis can either be a sign for an active microglia-driven process or alternatively occur in response to any neighboring pathological events. Therefore, any microgliosis in a patient specimen requires further examination.

Of note, the staining procedure including the selection of antibody clone, thickness of the section and the incubation times may affect the number of cells labelled. In mice, the exact hygiene status of an animal facility highly influences the number of cells observed. Consequently, the comparison with age-matched controls is essential.

### Step 3: Cell shape

As brain-resident macrophages, microglia usually have a ramified, spider-like shape. In the homeostatic CNS, microglia mostly present with a small soma and multiple processes (Fig. 3, Step 3). The processes are thin, also at the junction with the cell body. There are numerous ramifications. The thickness of the arms hardly changes in the course, making them look like a line. Upon activation, microglia rapidly change their morphology within a few minutes. Typically, the microglial somata appear bigger and the arms are thicker. Occasionally, the processes taper to a point. In this case, they have a bigger diameter at the soma and a smaller diameter in the periphery. This phenotype has previously been described as “thorny” or “spiky”. The processes can be shorter with less ramifications (Supplementary Fig. 2b). Thus, the whole cell may occupy a smaller area. Altogether, activated microglia cells look less delicate and more condensed. Light microscopy is very well suited for the assessment of the microglial morphology. Quantifiable morphometrical data (e.g. dendrite length, number of segments, number of branch points, cell volume) can be obtained by fluorescent immunohistochemistry, confocal microscopy and 3D reconstruction [11].

During homeostasis, microglia are considered to be long-lived in both mouse and man. They show only a small self-renewal rate through proliferation in the adult at a rate of 0.5% [1, 71]. There is no infiltration of circulating immune cells into the healthy CNS parenchyma. In contrast, myeloid cells cross the blood–brain barrier (BBB) and enter the CNS together with lymphocytes under neuroinflammatory conditions (e.g. in patients with active multiple sclerosis). Like parenchymal microglia, infiltrating parenchymal myeloid cells such as monocytes are also Iba1-positive. After infiltration into the brain parenchyma, bone marrow-derived macrophages initially retain their round shape, which allows them to be identified at this stage. Over time, the cells acquire a phenotype highly resembling brain-resident microglia. This phenotype includes transcriptional and morphological features. Of note, after ablation of microglia in mice, the microglial compartment is reconstituted by proliferation of CNS-resident cells and independent from bone-marrow derived precursors [4].

Iba1-positive cells with a round shape may point towards an infiltration of hematopoietic cells. Immunohistochemistry with markers for leucocytes, for example CD3 for T cells and CD20/B220 for B cells, might be useful to investigate this in more detail. However, fully activated microglia may also appear roundish and foamy and can no longer be distinguished from infiltrating Iba1^+^ monocytes.

### Step 4: Distribution pattern

The distribution pattern of microglia should be carefully examined considering several aspects. First, are the microglial features observed homogenous in the whole tissue section? Occasionally, the microglial phenotype differs regionally within the same section. For instance, white matter microglia may present with more activated features than grey matter microglia [61] (Fig. 3, step 4). Perivascular microglia accumulation might point towards a vascular pathology.

Second, do the individual microglia respect each other’s territory? In a physiological brain, the distance between a microglial cell and the surrounding microglia is comparatively constant; spots with two or more microglia cells accumulating are fairly rare (Fig. 3, step 4, upper left). Vice versa, finding this pattern commonly points towards pathological processes (Supplementary Fig. 2e). Iba1-positive structures with many microglia cells accumulating and indistinguishable cell borders are called microglia nodules. They are commonly found in chronic inflammatory conditions, including viral infections or putative autoimmune diseases, such as multiple sclerosis [65]. In particular, microglia nodules have been described in the context of HIV and COVID-19 encephalopathy [38, 62].

### Step 5: Distinct microglial phenotypes

Next, the researcher should look for rare but pretty distinct microglial phenotypes. Among these are the microglial nodules discussed earlier [35, 65] (Fig. 3, step 5). An increased Iba1^+^ cell density (see “Step 2: cell density”) can primarily result from higher numbers of invading blood-borne cells or an increase in self-renewal by proliferation. In the latter instance, it may be possible to detect microglia cells that are in the process of division [13]. The most reliable evidence is certainly the presence of mitotic figures within microglia cells. However, due to the short window within the cell cycle, mitotic figures can only be detected rarely. For this reason, it can be useful to combine Iba1 immunohistochemistry with the proliferation marker Ki-67 [18]. Even beyond the slightly longer time window of Ki-67 positivity, there may be signs of previous cell divisions. Thus, two closely located microglia cells with a connecting cytoplasmic bridge strongly suggests that the cells arose from a single cell that has been dividing.

Small bulgings on thin microglia processes, named knot-like structures, were demonstrated in brain samples from patients with hereditary diffuse leukoencephalopathy with spheroids (HDLS) [70]. The disease is characterized by various neurological symptoms including dementia. It is caused by different mutations in the *CSF1R* gene. Since the gene is predominantly expressed in microglia in the brain, the disease is considered a “primary microgliopathy” [55, 56].

Foam cells are transformed macrophages whose cytoplasm appears foamy and bubbly because of previously phagocytosed material, primarily lipids [33]. In the periphery, the formation of foam cells has been studied well in the context of atherosclerosis [33]. Foam cells can also be found within the CNS (Supplementary Fig. 2 h). They are typically found in multiple sclerosis lesions [21, 36]. In this case, the foam cells may also contain myelin [21]. The degradation of the myelin components follows a predictable temporal sequence. Thus, the chemical profile of the myelin degradation products can be used as a precise marker for the time dependent evolution of a lesion [3]. As a sign of an active debris clearance, foam cells are occasionally observed in close proximity to brain tumors or CNS abscesses.

Under certain neurodegenerative conditions in the human brain and in inactive lesions of multiple sclerosis the global number (density) of microglia is reduced. This appears to be a consequence of microglia senescence during active disease [23, 68]. Microglia senescence is characterized by clumping and loss of cell processes finally resulting in cell death by apoptosis. Senescent microglia can be visualized by immunohistochemical staining for ferritin, since it is associated with microglia iron load [23]. Senescent microglia may be a result of oxidative injury and may be one of the reasons for microglia dysfunction in the cortex of patients with Alzheimer’s disease.

### Step 6: Interaction with other cell types and structures

Finally, when assessing microglia using light microscopy, the examiner should look for any signs of excessive interactions and/or physical contacts with other cell types or structures. In the case of close physical contact with neurons, the phagocytosis of neurons by microglia, so-called neuronophagy, could be observed (Fig. 3, step 6). The interaction with oligodendrocytes is of special interest, in particular regarding inflammatory demyelinating disorders such as MS. The Luxol-Fast-Blue-Periodic-Acid-Schiff (LFB-PAS) stain can help to find demyelinating plaques. Moreover, small cells containing vibrant blue-stained myelin fragments demonstrate myeloid cells that are actively phagocyting white matter components. Iba1-positive cells are also found after ischemic events: the histopathological findings in a subacute ischemic brain infarct, phase II, includes the infiltration of macrophages [76].

In neurodegenerative disorders characterized by the formation of Aβ deposits, plaque-associated microglia can be present [27].

Both microglia and macrophages are known for colonizing CNS neoplasms, as for example gliomas. The so-called tumor-associated macrophages (TAMs) have been carefully characterized and their therapeutic potential is currently being explored [22].

## Novel cell markers for microglia in the healthy and diseased CNS

After purification using a 37% Percoll gradient, microglia can be identified by flow cytometry with leukocyte common antigen (CD45) and integrin alpha M (ITGAM, CD11b) as commonly used markers [7, 36]. These markers can also be used to target CNS-associated macrophages (CAMs, e.g. perivascular macrophages) if the tissue has been digested enzymatically before [26]. Cells positive for lineage markers (T-cell surface glycoprotein CD3, B-lymphocyte antigen CD19, B-lymphocyte antigen CD20) should ideally be excluded.

**Fig. 4:**
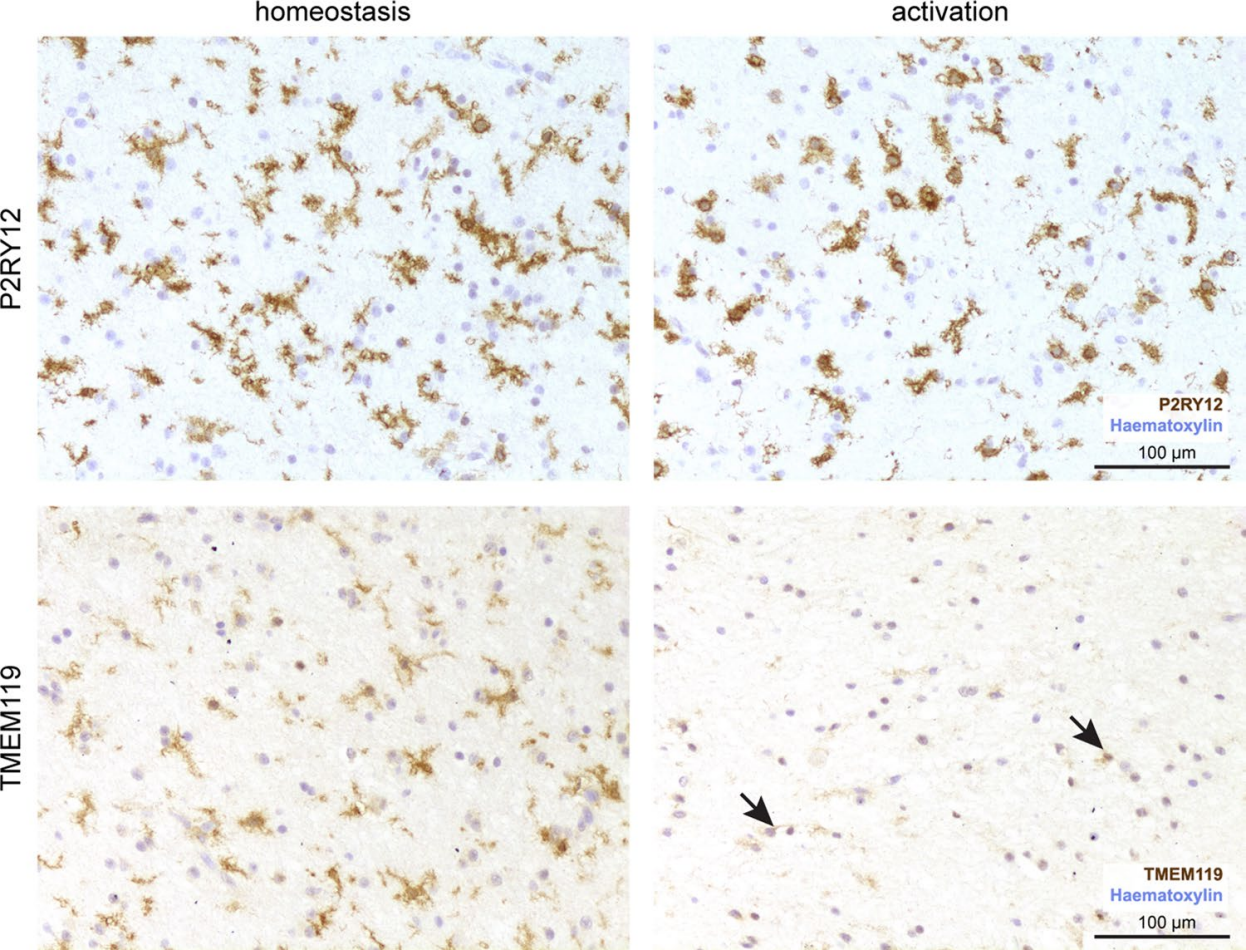
Spatial heterogeneity of the myeloid compartment within a single tissue section of a patient with encephalitis revealed by P2RY12 and TMEM119 immunohistochemistry. The immunohistochemical reactions for P2RY12 (brown, upper panel) and TMEM119 (brown, lower panel) are shown in two regions of the same tissue section of a patient with encephalitis. The microglia cells (as identified by P2RY12) positivity show a downregulation/loss of TMEM119 upon activation. Counterstaining with haematoxylin (blue). Scale bar: 100 µm

With its spatial resolution and the possibility of assessing the cell morphology, immunohistochemistry is particularly well suited for the analysis of microglia. As discussed earlier, the immunohistochemical reaction against Iba1 labels brain-resident microglia, CAMs as well as blood-borne myeloid cells that invade the brain parenchyma under certain conditions. Transmembrane protein 119 (TMEM119) [2] and P2Y purinoceptor 12 (P2RY12) [6] were reported as novel markers that help to discriminate between microglia, CAMs and infiltrating myeloid cells. Both markers were shown to be expressed on microglia, but not on CAMs and infiltrating monocytes. Therefore, a cell within the brain parenchyma with positivity for TMEM119 or P2RY12 can be unambiguously identified as microglia cell (Fig. 4). P2RY12 is an excellent marker for the homeostatic phenotype of microglia and is rapidly lost in many pathologies. Although TMEM119 is also downregulated upon activation, this process is incomplete and often takes more time. Both markers have been used in a detailed phenotypic characterization of microglia in multiple sclerosis lesions [79]. Thus, TMEM119 is a good marker for microglia in initial and early lesions, but becomes less useful with lesion maturation (Fig. 4). Further microglia characterization was then achieved by the use of markers, which define specific functional states. As examples, the expression of MHC Class I or II antigens or CD86 is related to antigen presentation, CD68 to phagocytosis, iNOS and molecules of the NOX-2 complex to the production of nitric oxide and oxygen intermediates, and Fc- and complement receptors to the uptake of opsonized tissue elements. Ferritin indicates iron loading. Novel CAM-specific markers are supplementing the technical repertoire. The gene *Mrc1* encoding for the mannose receptor (CD206), for instance, is expressed on CAMs and barely on microglia. Perivascular macrophages, for instance, also express the scavenger receptor cysteine-rich type 1 protein M130 (CD163), a receptor for hemoglobin–haptoglobin complexes [29]. While homeostatic microglia show no expression of CD163, it has been shown that parenchymal myeloid cells can upregulate CD163 under certain conditions [79]. Commonly used markers are summarized in Table 1.

**Table 1:** Commonly used immunomarkers for the characterization of different microglial states in the healthy and diseased murine and human CNS are shown

Epitope	Description	Usage	Recommended antibody
CD11b	Commonly used for flow cytometry (in combination with CD45) for the isolation of microglia cells/CAMs	FC	Human: Biolegend, cat. # 301310, 1:20 Mice: eBioscience, cat. # 17-0112-83, 1:200
CD163	Marker for perivascular macrophages. Parenchymal microglia may upregulate CD163 upon activation	IHC	Novus, cat. # NB110-40686, 1:100
CD206	Marker for perivascular macrophages	IHC	BioRad, cat. # MCA2235, 1:100
CD45	Commonly used for flow cytometry (in combination with CD11b) for the isolation of microglia cells/CAMs	FC	Human: Biolegend, cat. # 304008, 1:20 Mice: eBioscience, cat. # 12-0451-83, 1:200
CD68	Marker for lysosomal activity	IHC	Human: DAKO, cat. # M0876, 1:400 Mice: BioRad, cat. # MCA1957, 1:100
F4/80	Commonly used myeloid marker in mice	IHC	BioRad, cat. # MCA497GA, 1:100
Iba1	Pan-myeloid marker labelling microglia, perivascular macrophages, meningeal macrophages and infiltrating myeloid cells	IHC	abcam, cat. # ab178846, 1:1000
Mac-3	Marker for lysosomal activity	IHC	Becton Dickinson, 553322, 1:400
MHC-II	Antigen-presenting cells	IHC	Human: Dako, cat. # M0775, 1:400 Mice: Abcam, cat. # ab23990, 1:400
P2RY12	Marker for microglia cells; absent in perivascular macrophages or meningeal macrophages. Of note, parenchymal microglia cells may downregulate P2RY12 upon activation. Consequently, a ramified P2RY12-positive cell in the brain parenchyma can be identified as a microglia cell.	IHC	Anaspec, Cat. # AS-55043A, 1:200
Pu.1	Nuclear pan-myeloid marker	IHC, FC	Cell signaling technology, cat. # 2258S, 1:200
SLC2A5	Glucose transporter type 5 labelling parenchymal myeloid cells	IHC	Abcam, Atlas Antibodies, HPA005449, 1:250
TMEM119	Marker for microglia cells; absent in perivascular macrophages or meningeal macrophages. Of note, parenchymal microglia cells may downregulate TMEM119 upon activation. Consequently, a ramified TMEM119-positive cell in the brain parenchyma can be identified as a microglia cell.	IHC	Human: Abcam, cat. # ab185333, 1:250 Mice: Abcam, cat. # ab209064, 1:500

## Transcriptional/epigenetic changes and chromatin accessibility in microglia

**Fig. 5:**
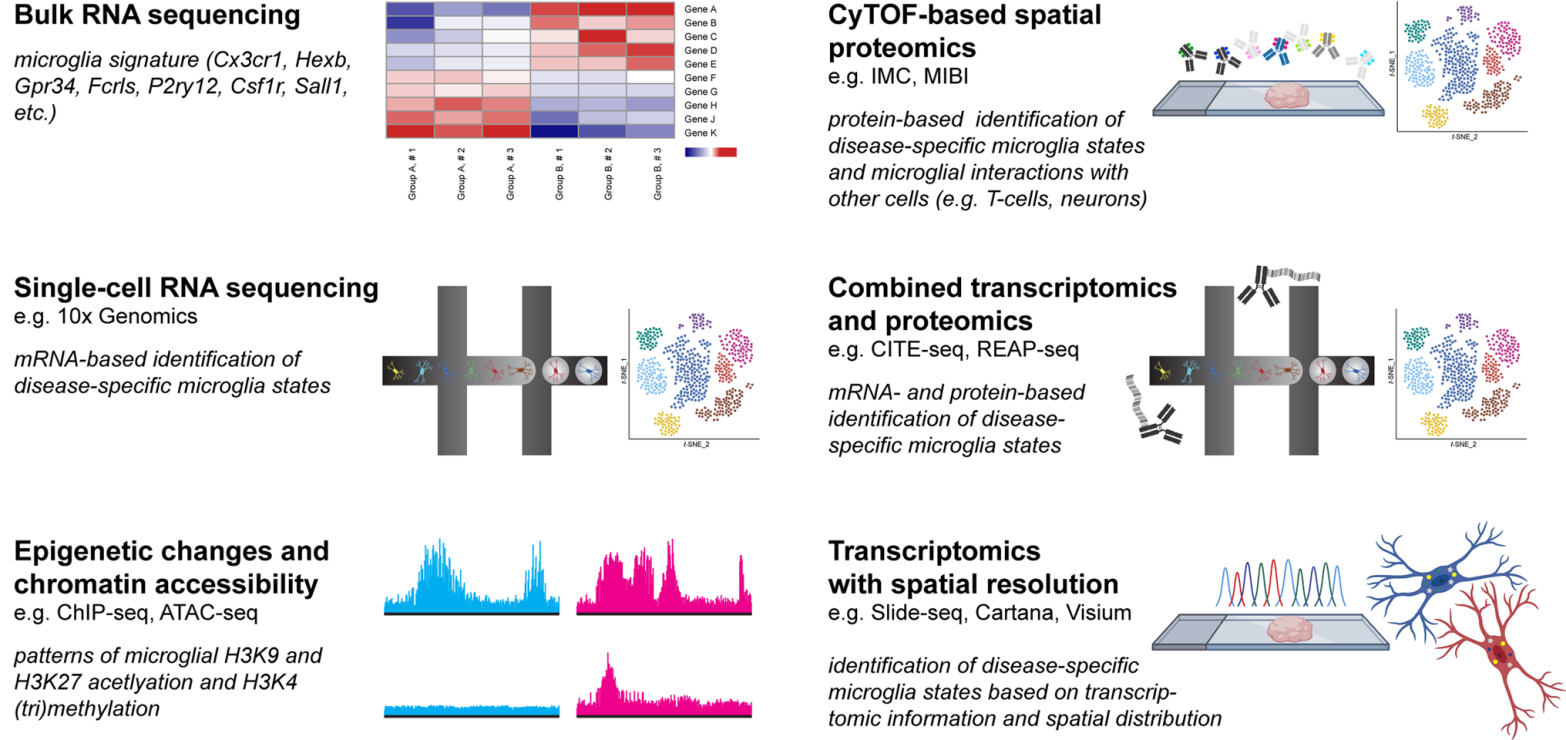
Novel high-throughput technologies expand the diagnostic spectrum of research on microglia. Several emerging cutting-edge methods for studying the microglial phenotype are depicted in a graphical manner. The pictograms demonstrate a gene expression heatmap (Bulk RNA sequencing), a droplet-based analysis of single cells with subsequent *t*-SNE visualization (Single-cell RNA sequencing), the chromatin landscape using ATAC-seq (epigenetic changes and chromatin accessibility), an objective slide incubating with metal-coupled antibodies with subsequent single-cell analysis (CyTOF-based Imaging Mass Cytometry), barcoded antibodies in combination with droplet-based microfluidics system (combined transcriptomics and proteomics) and a tissue section analyzed by in-situ-sequencing as well as two microglia cells with different intracellular symbols representing different genes (transcriptomics with spatial resolution)

During the last decade, the microglial transcriptome has been studied extensively in mice and humans under both homeostatic and disease conditions [19, 32]. Despite minor differences across different studies, there is a unique microglial core gene signature that is partially overlapping with other tissue macrophages outside the CNS. Among those, genes encoding for transcription factors (e.g. *Sall1* [32]), (cell surface) receptors (*Cx3cr1*, *Gpr34*, *Fcrls* [32]*, P2ry12, Csf1r*), and enzymes (*Hexb*) have been described (Fig. 5). Of note, some genes are downregulated upon activation, for example P2RY12 [6] as described above. Microglia cells can be identified by their distinct transcriptomic signature [20]. Histone modifications (e.g. H3K9 and H3K27 acetylation [9, 32] or H3K4 (tri)methylation [32, 37, 45]) can be identified by chromatin immunoprecipitation DNA-sequencing (ChIP-Seq). Those histone modifications may lead to enhanced chromatin accessibility, ultimately influencing the cell’s transcriptome [37]. An assay of transposase-accessible chromatin and subsequent sequencing (ATAC-seq) [5, 45] is commonly used to investigate the genome-wide chromatin accessibility.

## Assessing microglia heterogeneity on the single-cell level

While conventional imaging methods suggest that microglia form a comparatively homogenous population, recent studies demonstrated that microglia are heterogeneous. By using single-cell RNA-sequencing technology, microglia can be divided into distinct (disease-specific) states or subclusters [26, 27, 36, 48]. The gene expression pattern of the disease-associated clusters is of particular interest. Since novel, therapeutic targets may only be expressed on cells of a certain cluster, they can easily be missed when analyzing all microglia as a whole. Consequently, the analysis on the single-cell level is a promising approach in microglia research. So far, many CNS pathologies and their respective animal models have been analyzed using single-cell RNA sequencing approaches, e.g. Alzheimer’s disease [27, 48], Multiple Sclerosis [26, 36] or gliomas [12]. Intracerebral macrophages other than microglia, i.e. perivascular macrophages, meningeal macrophages and choroid plexus macrophages are commonly summarized as CAMs [26] or border-associated macrophages (BAMs) [43]. Single-cell technologies have tremendously helped to better understand CAMs/BAMs in health and disease [26, 43, 73]. Single-cell RNA-sequencing was able to define myeloid cell clusters in isocitrate dehydrogenase (IDH) 1 mutant astrocytomas that were not present in IDH-1 wild-type gliomas, which may have both diagnostic and therapeutic consequences [12].

## Novel techniques in microglia research

New high-throughput technologies continuously complement the existing spectrum of experimental methods, in particular in the field of proteomics [63], metabolomics [72] and lipidomics [46] (Fig. 5). Cytometry by time-of-flight (CyTOF) allows the simultaneous investigation of more than forty markers in single-cell suspensions, strongly expanding the possible number of channels comparted to conventional flow cytometry [43]. CyTOF-based techniques, e.g. imaging mass cytometry (IMC) or multiplexed ion beam imaging (MIBI), add spatial information to single-cell data at protein level [62]. Using IMC together with clinical data such as long-term survival, new subgroups of disease entities could be identified [25]. Moreover, this technique has recently been used to characterize the immune landscape in the brainstem of patients who died of COVID-19 [62].

Furthermore, novel techniques combining different analyses are emerging, e.g. cellular indexing of transcriptomes and epitopes by sequencing (CITE-seq) or RNA expression and protein sequencing (REAP-seq) linking transcriptomics and proteomics [17]. Different subsets of tumor-associated macrophages in glioblastomas could be identified by applying CITE-seq [51]. In-situ sequencing (ISS) or in-situ capture approaches (e.g. the Cartana or Visium platform) aim to link transcriptomic information to spatial distribution [8, 39]. Another RNA sequencing technique with spatial resolution is Slide-seq that was used to characterize traumatic brain injury in mice [58].

## Chances and limitations of the new technologies

The amount of data obtained through the use of novel high-throughput methods is huge and data analysis is quite complex. However, to what extent the findings from animal models can be transferred to the situation in a human being is a recurring question. In this regard, especially three aspects are important to note. First, in spite of a common core gene expression signature that is preserved across evolution, microglia from animals and humans show distinct differences in gene expression and (as a consequence) presumably in cell function. Secondly, the genetic basis is more diverse in human individuals compared to inbred mice. It is therefore conceivable that some individuals show a certain predisposition for enhanced microglia activation. This hypothesis is supported by the clinical course of microgliopathies. Microgliopathies form a group of hereditary diseases in which a gene mutation in myeloid cells leads to neurodegenerative symptoms in particular. Studying those rare entities might lead to a better understanding of microglia-driven brain pathologies in general. Thirdly, it has been shown that microglia are both reacting quickly and acting in a long-term manner, likely due to an epigenetic reprogramming of the cells [75]. Thus, an observed microglial phenotype can be caused by any prior alteration of brain homeostasis. This means that comorbidities, medications, former and current infections and any other environmental factor throughout life have to be taken into account when analyzing tissue specimens. While those factors should be well controlled in animal models, choosing the right control group for human samples can be challenging.

All novel methods come with their very own challenges and hurdles. Those challenges can be of a technical (e.g. low RNA sequencing depth) or conceptual nature (e.g. high gene expression pattern does not necessarily lead to higher protein levels). Another issue is the tissue availability. Many techniques do require specific tissue preservation protocols other than formaldehyde-fixed paraffin-embedded tissue (FFPE). Novel technologies such as the CyTOF-based Imaging Mass Cytometry also use FFPE samples, granting access to biobanks with archival human material. This technique has recently been used to examine the changes within the CNS after SARS-CoV-2 infection [62]. Using FFPE tissue, this method may connect cutting-edge technology with daily pathological routine diagnostics.

Despite all technical progress, research projects using sophisticated novel technologies do need a clear experimental design and research strategy. Respective investigations have to be performed with a clearly defined research plan. The human material should be classified perfectly and stored adequately. Proper controls including suitable disease controls must be included. Therefore, the collection of an optimal patient cohort remains a major challenge when studying CNS pathologies.

## Outlook

Since microglia rapidly react to even subtle alterations in CNS homeostasis, they can be seen as sensors for neurological dysfunctions and/or disorders. Many of them are far from being understood completely. Investigating microglia in the disease context might lead to a better understanding of the respective disease itself. Given their ability to modulate CNS pathologies, studying microglia possibly creates new therapeutic options as well [52]. Vice versa, the absence of microglia alterations strongly indicates the absence of any neuropathology.

In this review, we have summarized the technical possibilities for analyzing microglia phenotypes in tissue specimens obtained from humans and animals. We have proposed a simple step-by-step protocol for analyzing microglia phenotypes in histology. It should enable researchers of any specialty to evaluate the microglial phenotype in any given tissue sample. Furthermore, it should provide ideas for more detailed, subsequent experiments.

So far, virtually all available techniques for the analysis of microglia cells rely on tissue: either as a whole (e.g. for histological analyses) or processed (e.g. as a single-cell suspension for single-cell RNA sequencing). This is not a major constraint for the analysis of animal models or post-mortem autopsy cases. Contrarily, it is a limitation in patients with a suspected brain pathology. With very good reason, CNS biopsies are not taken carelessly or casually. However, not having a biopsy specimen implies not knowing anything about the microglial phenotype that could inform about the underlying (or even a just developing) pathology. Therefore, the development of non-invasive techniques for the analysis of the microglial phenotypes might close the gap. As such, PET imaging with microglia-specific radiotracers in the future sounds tempting. Detecting disease-specific microglia metabolites in liquid biopsies might be another promising approach.

Taken together, the technical possibilities for studying microglia have evolved rapidly in recent years. New methods provided unprecedented insights into microglia biology in general as well as into the etiology and course of many CNS disorders. Despite of all the progress made, the potential of microglia has certainly not yet been fully exploited. Many more exciting years in microglia research are yet to come.

## Supplementary Information

Below is the link to the electronic supplementary material.

Supplementary Figure 1: Exemplary step-by-step protocol for performing an immunohistochemistry on human or murine tissue sections as commonly performed at the Institute for Neuropathology at the University Medical Center Freiburg. The protocol for the preparation of cryopreserved sections (upper left) or FFPE sections (upper right) is shown. Below, the steps for fluorescent (lower left) and chromogenic (lower right) immunohistochemistries (e.g. for Iba1, TMEM119 or P2RY12) are explained. Supplementary Figure 2: Different microglia/macrophage morphologies are depicted in exemplary patient samples. The immunohistochemistry for Iba1 (brown) is exemplarily shown in different CNS pathologies. Counterstaining with haematoxylin (blue). Scale bar: 100 µm. a: CNS myeloid cells first need to be identified based on the anatomical location: a haematopoetic Iba1-positive cell can be observed within a blood vessel (asterisk). Within the meninges, meningeal macrophages are labelled by Iba1 (blue arrows). Perivascular macrophages present with an elongated shape and less ramifications compared to microglia (green arrows). The density of parenchymal microglia (black arrows) appears normal. The cells are ramified and do not show a spiky phenotype. The distribution pattern appears regular with the cells respecting each other’s territory. No distinct microglial phenotypes are observed. Moreover, there is no excessive interaction with other cell types. In sum, the microglial phenotype appears homeostatic. b: The cell density in sample b is comparable to sample a. Microglia with ramifications can be found (arrows). In some areas, small parenchymal Iba1-positive cells with less protrusions are visible, resembling an activated phenotype. Nevertheless, the cells do respect each other’s territory. c: Iba1-positive cells with a characteristic “spiky” morphology are seen (arrows). d: A similar phenotype with “thorny” cells can be observed in d. Some cells appear to have less protrusions (asterisks). e: Spatial differences can be identified in e. The cells in closer vicinity to the lesion (blue arrow) look more condensed compared to more distant microglia (green arrow) showing fine protrusions. The cells even closer to the lesion do no longer have any processes. They show overlapping territories. f: The macrophages in the glioblastoma specimen in f are comparatively large in size (arrows). g: Some ramified cells can be found in sample g (arrow). A large number of Iba1-positive cells appears small and round, indicating haematopoetic cells. h: Iba1-positive haematopoetic cells are also found within blood vessels in h (asterisks). A perivascular macrophage can be identified (blue arrow). A few cells show a slightly ramified morphology (green arrow). Many cells have undergone foam cell transformation (black arrows), indicating an active debris clearance. (PDF 7066 KB)
