# Supplementary figures and images for: Analyzing microglial phenotypes across neuropathologies: a practical guide
Source: Acta Neuropathol. 2021 Oct 8;142(6):923–36. doi: 10.1007/s00401-021-02370-8 (PMC8498770; doi:10.1007/s00401-021-02370-8)

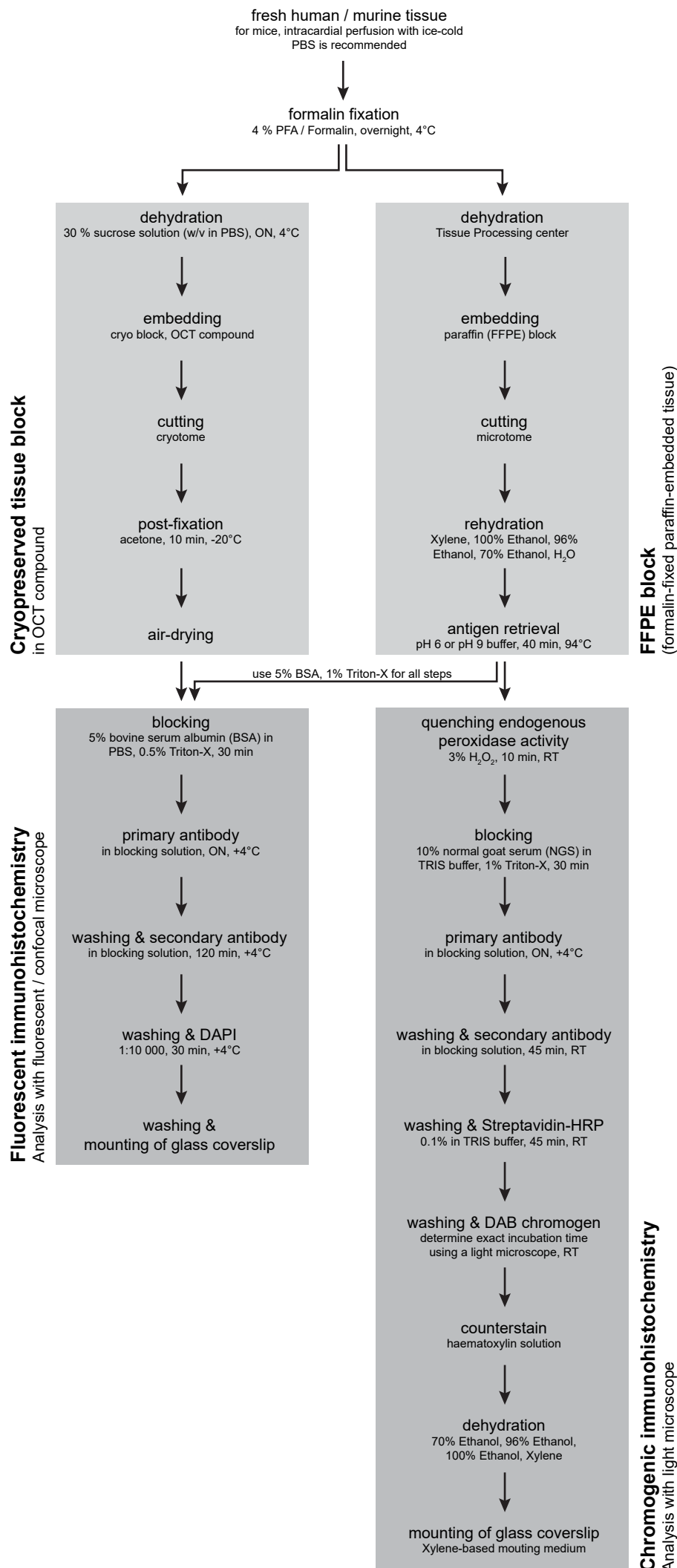

**Supplementary Figure 1**

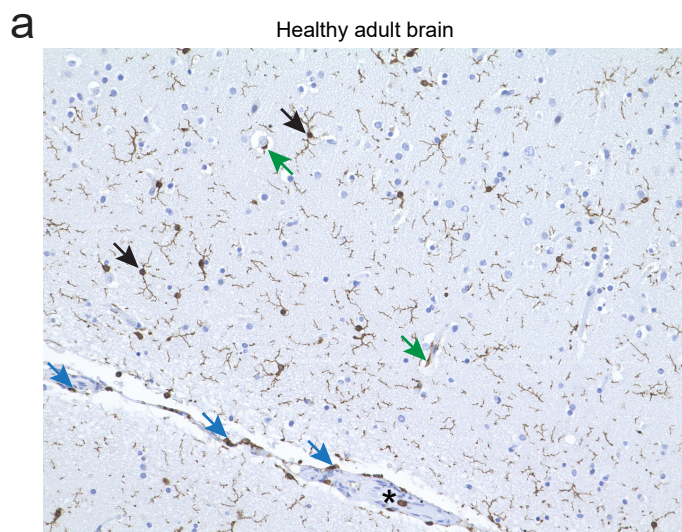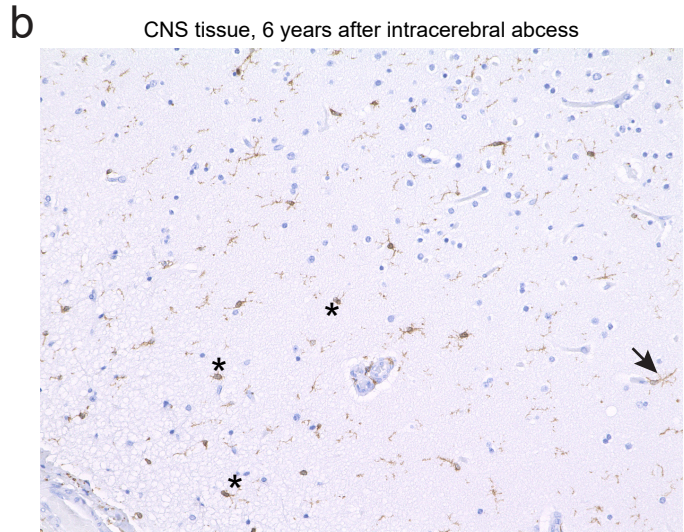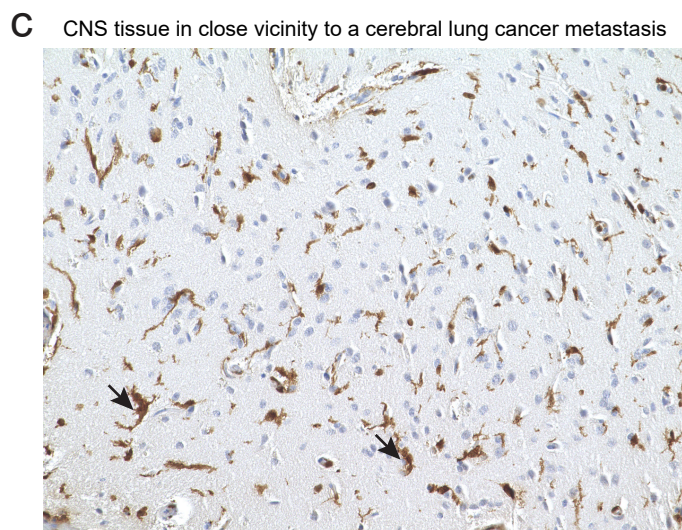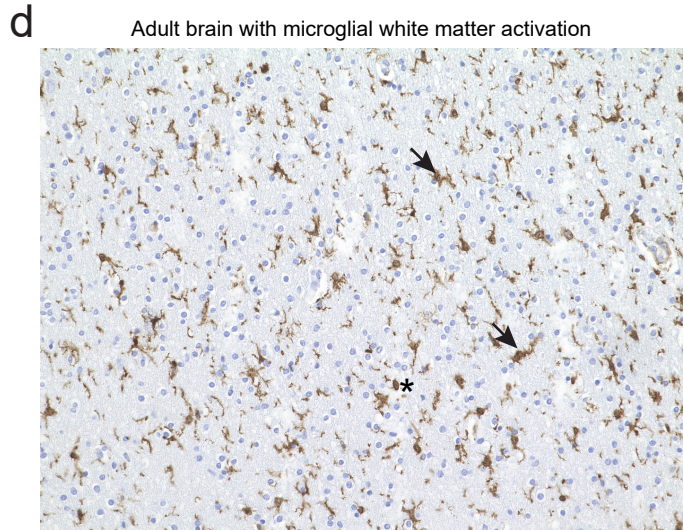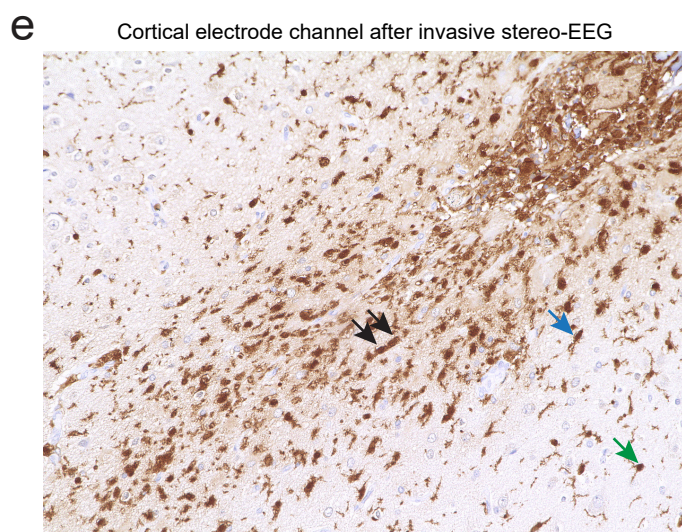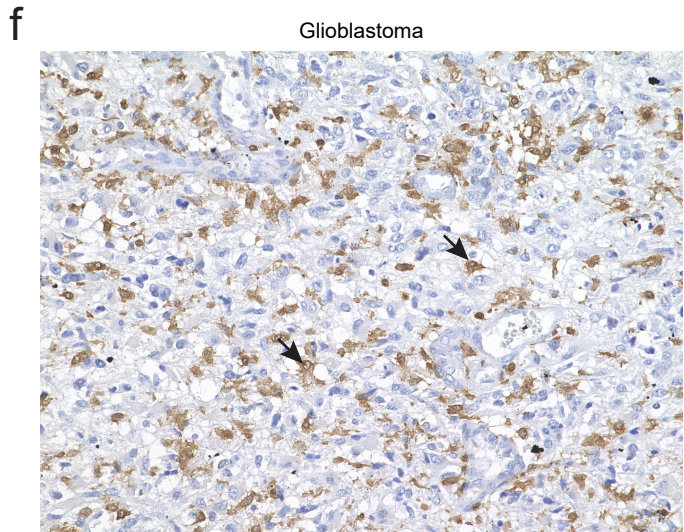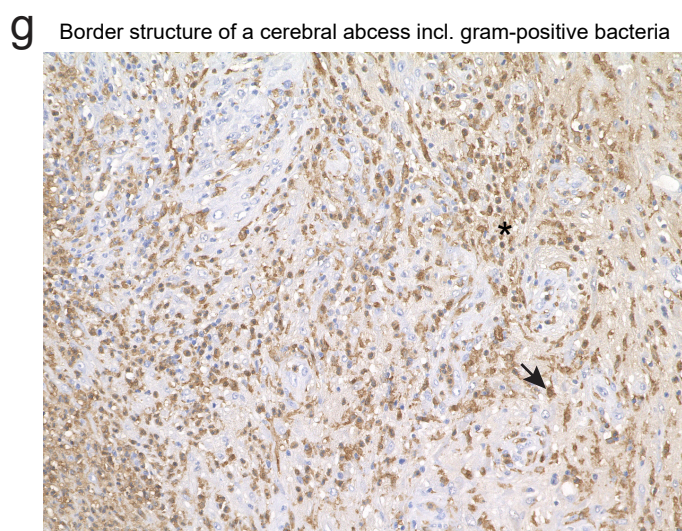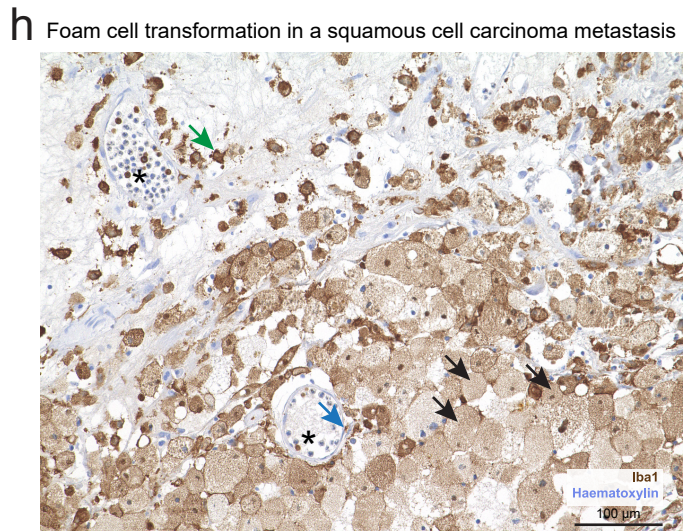

**Supplementary Figure 2**

Supplement: Supplementary file 1 — Supplementary Figure 1: Exemplary step-by-step protocol for performing an immunohistochemistry on human or murine tissue sections as commonly performed at the Institute for Neuropathology at the University Medical Center Freiburg. The protocol for the preparation of cryopreserved sections (upper left) or FFPE sections (upper right) is shown. Below, the steps for fluorescent (lower left) and chromogenic (lower right) immunohistochemistries (e.g. for Iba1, TMEM119 or P2RY12) are explained. Supplementary Figure 2: Different microglia/macrophage morphologies are depicted in exemplary patient samples. The immunohistochemistry for Iba1 (brown) is exemplarily shown in different CNS pathologies. Counterstaining with haematoxylin (blue). Scale bar: 100 µm. a: CNS myeloid cells first need to be identified based on the anatomical location: a haematopoetic Iba1-positive cell can be observed within a blood vessel (asterisk). Within the meninges, meningeal macrophages are labelled by Iba1 (blue arrows). Perivascular macrophages present with an elongated shape and less ramifications compared to microglia (green arrows). The density of parenchymal microglia (black arrows) appears normal. The cells are ramified and do not show a spiky phenotype. The distribution pattern appears regular with the cells respecting each other’s territory. No distinct microglial phenotypes are observed. Moreover, there is no excessive interaction with other cell types. In sum, the microglial phenotype appears homeostatic. b: The cell density in sample b is comparable to sample a. Microglia with ramifications can be found (arrows). In some areas, small parenchymal Iba1-positive cells with less protrusions are visible, resembling an activated phenotype. Nevertheless, the cells do respect each other’s territory. c: Iba1-positive cells with a characteristic “spiky” morphology are seen (arrows). d: A similar phenotype with “thorny” cells can be observed in d. Some cells appear to have less protrusions (asteri [file 401_2021_2370_MOESM1_ESM.pdf]
